# Ethyl 2-[({[4-amino-5-cyano-6-(methyl­sulfan­yl)pyridin-2-yl]carbamo­yl}meth­yl)sulfan­yl]acetate monohydrate

**DOI:** 10.1107/S1600536814012495

**Published:** 2014-06-04

**Authors:** Mehmet Akkurt, Joel T. Mague, Shaaban K. Mohamed, Bahgat R. M. Hussein, Mustafa R. Albayati

**Affiliations:** a Department of Physics, Faculty of Sciences, Erciyes University, 38039 Kayseri, Turkey; b Department of Chemistry, Tulane University, New Orleans, LA 70118, USA; c Chemistry and Environmental Division, Manchester Metropolitan University, Manchester M1 5GD, England; d Chemistry Department, Faculty of Science, Minia University, 61519 El-Minia, Egypt; e Chemistry Department, Faculty of Science, Sohag University, 82524 Sohag, Egypt; f Kirkuk University, College of Science, Department of Chemistry, Kirkuk, Iraq

## Abstract

The title compound, C_13_H_16_N_4_O_3_S_2_·H_2_O, crystallizes in a ‘folded’ conformation with the ester group lying over the carbamoyl moiety, with one solvent water mol­ecule. The mol­ecular conformation is stabilized by an intra­molecular C—H⋯O hydrogen bond, and an N—H⋯O hydrogen-bonding inter­action involving the lattice water mol­ecule. The packing involves N—H⋯N, N—H⋯O, O—H⋯N and O—H⋯O hydrogen bonds and consists of tilted layers running approximately parallel to the *c* axis, with the ester groups on the outer sides of the layers and with channels running parallel to (101).

## Related literature   

For the synthesis of amino-cyano pyridines, see: Shi *et al.* (2005 ▶). For pyridines as inter­mediates in the synthesis of different heterocyclic compounds, see: Konda *et al.* (2010 ▶). For the pharmaceutical activity of functionalized pyridine derivatives, see: Dorigo *et al.* (1993 ▶); Dolle *et al.* (1995 ▶); Murata *et al.* (2003 ▶). For industrial applications of pyridine compounds, see: Lohray *et al.* (2004 ▶); Merja *et al.* (2004 ▶); Chaki *et al.* (1995 ▶); Thomae *et al.* (2007 ▶). For hydrogen-bond motifs, see: Bernstein *et al.* (1995 ▶).
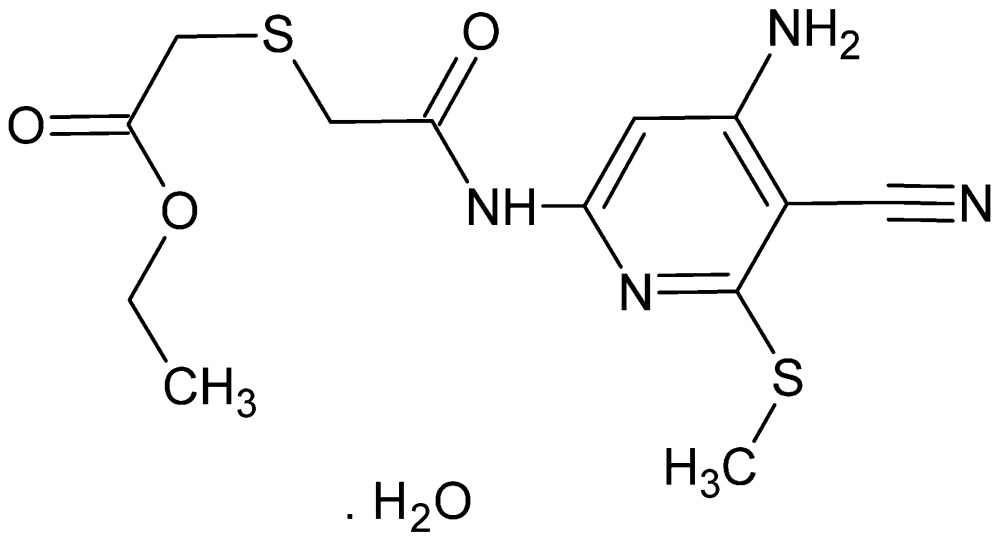


## Experimental   

### 

#### Crystal data  

C_13_H_16_N_4_O_3_S_2_·H_2_O
*M* *_r_* = 358.45
Triclinic, 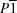

*a* = 9.0806 (12) Å
*b* = 9.2444 (12) Å
*c* = 10.7856 (14) Å
α = 101.843 (2)°
β = 100.1750 (19)°
γ = 105.9480 (19)°
*V* = 825.48 (19) Å^3^
*Z* = 2
Mo *K*α radiation
μ = 0.35 mm^−1^
*T* = 150 K
0.26 × 0.26 × 0.12 mm

#### Data collection  

Bruker SMART APEX CCD diffractometer
Absorption correction: multi-scan (*SADABS*; Bruker, 2013 ▶) *T*_min_ = 0.83, *T*_max_ = 0.96
15313 measured reflections
4292 independent reflections
3773 reflections with *I* > 2σ(*I*)
*R*_int_ = 0.034

#### Refinement  

*R*[*F* ^2^ > 2σ(*F* ^2^)] = 0.037
*wR*(*F* ^2^) = 0.099
*S* = 1.05
4292 reflections
210 parameters
H-atom parameters constrained
Δρ_max_ = 0.44 e Å^−3^
Δρ_min_ = −0.37 e Å^−3^

### 

Data collection: *APEX2* (Bruker, 2013 ▶); cell refinement: *SAINT* (Bruker, 2013 ▶); data reduction: *SAINT*; program(s) used to solve structure: *SHELXT* (Sheldrick, 2008 ▶); program(s) used to refine structure: *SHELXL2014* (Sheldrick, 2008 ▶); molecular graphics: *DIAMOND* (Brandenburg & Putz, 2012 ▶); software used to prepare material for publication: *SHELXTL* (Sheldrick, 2008 ▶).

## Supplementary Material

Crystal structure: contains datablock(s) global, I. DOI: 10.1107/S1600536814012495/sj5406sup1.cif

Structure factors: contains datablock(s) I. DOI: 10.1107/S1600536814012495/sj5406Isup2.hkl

Supporting information file. DOI: 10.1107/S1600536814012495/sj5406Isup3.cml

CCDC reference: 1005734

Additional supporting information:  crystallographic information; 3D view; checkCIF report

## Figures and Tables

**Table 1:** Hydrogen-bond geometry (Å, °)

*D*—H⋯*A*	*D*—H	H⋯*A*	*D*⋯*A*	*D*—H⋯*A*
N3—H3*A*⋯N2^i^	0.91	2.43	3.3291 (18)	168
N3—H3*B*⋯O1^ii^	0.91	2.03	2.9345 (18)	177
N4—H4*A*⋯O4	0.91	2.01	2.9212 (16)	174
O4—H4*B*⋯N2^iii^	0.84	2.17	3.0032 (19)	172
O4—H4*C*⋯O2^iv^	0.84	2.09	2.9122 (18)	168
C4—H4⋯O1	0.95	2.25	2.8484 (17)	121

Symmetry codes: (i) 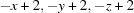; (ii) 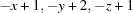; (iii) 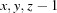; (iv) 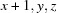.

**Fig. 1.**
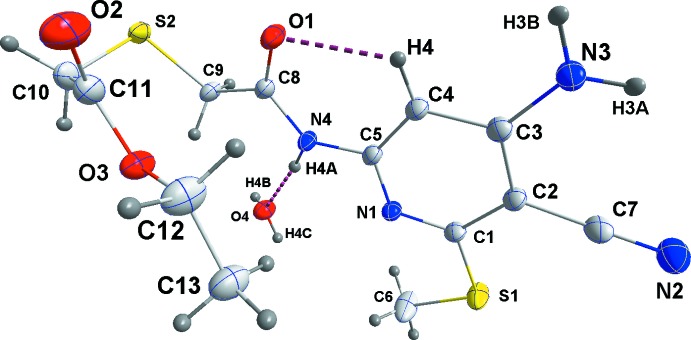
Perspective view of the asymmetric unit with 50% probability ellipsoids and hydrogen bonds depicted by dashed lines.

**Fig. 2.**
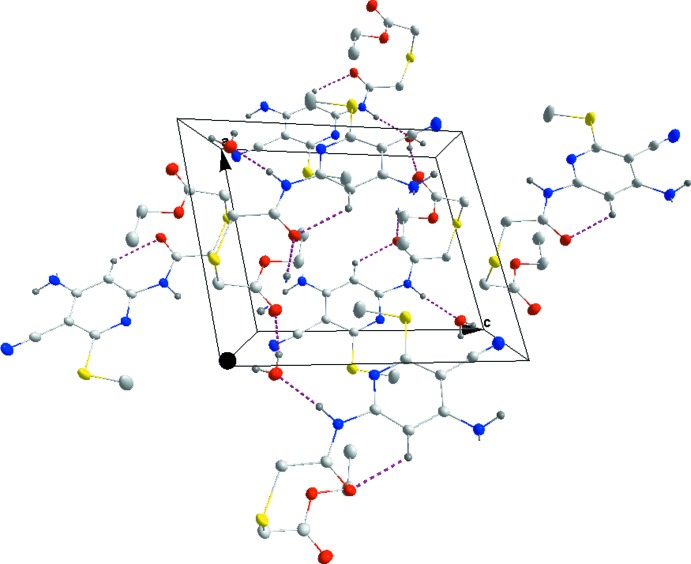
Packing projected down the b axis showing the inter- and intramolecular hydrogen bonds as dashed lines.

**Fig. 3.**
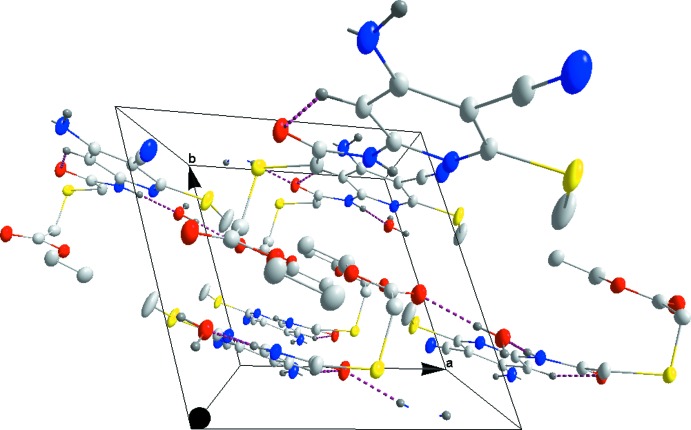
Packing projected along the c axis showing the tilted layers.

**Fig. 4.**
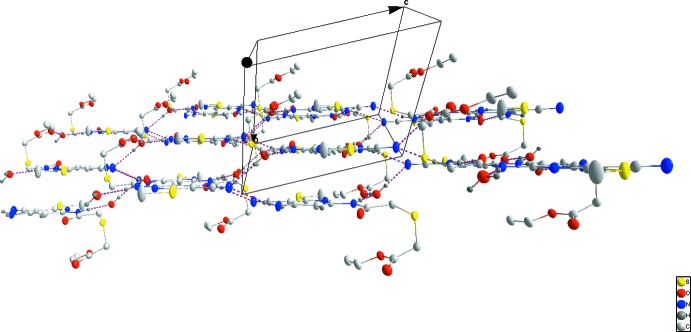
Packing viewed along the axis of the channels. Displacement ellipsoids for non-H atoms are drawn at the 50% probability level.

## supplementary crystallographic information

### S1. Comment

A great deal of interest has been focused on the synthesis of functionalized pyridine derivatives due to their biological activities (Shi *et al.*, 2005). For example, some 2-pyridine radicals are incorporated into the structures of cardiotonic agents such as milrinone (Dorigo *et al.*, 1993) and HIV-1 specific transcriptase inhibitors (Dolle *et al.*, 1995). Amino-cyanopyridines have been identified as IKK-β inhibitors (Murata *et al*., 2003). Many pyridine derivatives are of commercial interest being used as herbicides, fungicides, pesticides, and dyes (Lohray *et al.*, 2004; Merja *et al.*, 2004; Chaki *et al.*, 1995; Thomae *et al*., 2007). Besides, pyridine derivatives are important and useful intermediates in the preparation of a variety of heterocyclic compounds (Konda *et al.*, 2010). In view of these observations and in continuation of our work on the synthesis of heterocyclic systems for biological evaluations, we report here the synthesis and crystal structure of the title compound.

The title compound (Fig. 1) crystallizes in a "folded" conformation with the ester group lying over the carbamoyl moiety such that the dihedral angle between the best planes through the pyridyl ring and the C11–C13/O3 unit is 22.4 (1)°.

Molecular conformation is stabilized by an intramolecular C—H···O hydrogen bond, forming a S(6) motif, Fig. 1, (Bernstein *et al.*, 1995) and an N—H···O hydrogen bonding interaction involving the lattice water molecule.

This conformation appears to result from the several hydrogen bonding interactions involving the lattice water molecule, Fig. 2 and Table 1. The packing consists of tilted layers running approximately parallel to the *c* axis, Fig. 3, with the ester groups on the outsides of the layers and having channels running parallel to (101), Fig. 4.

### S2. Experimental

To a solution of *N*-[4-amino-5-cyano-6-(methylthio)pyridin-2-yl]-2-chloroacetamide (0.5 g, 1.95 mmol) in 30 ml ethanol and a few drops of triethylamine as a catalyst, ethyl mercaptoacetate (0.23 g, 1.95 mmol) was added. The reaction mixture was refluxed for 3 h at 350 K. The reaction mixture was allowed to cool down and the excess solvent was evaporated under reduced pressure. The precipitate which formed was filtered off, dried under vacuum and recrystallized from ethanol to furnish colourless crystals (yield 0.62 g; 95%). Mp. 423 – 425 K.

IR (ν_max_, cm^-1^): 3431, 3335, 3227, (NH_2_+NH), 2915 (CH aliph.), 2203 (C≡N), 1728 (C=O ester), 1641 (C=O amidic); ^1^HNMR (DMSO-d_6_), δ, p.p.m.: 10.34 (s, 1H, NH exchanged by D_2_O), 7.28 (s, 1H, CH pyridyl), 7.00 (s, 2H, NH_2_ exchanged by D_2_O), 4.11- 4.06(q, J = 8 Hz, 2H, CH_2_), 3.50 (s, 2H, CH_2_), 3.49 (s, 2H, CH_2_), 2.53 (s, 3H, CH_3_), 1.2–1.17 (t, J = 8 Hz, 3H, CH_3_).

### S3. Refinement

H-atoms attached to carbon were placed in calculated positions (C—H = 0.95 - 0.98 Å) while those attached to nitrogen were placed in locations derived from a difference map and their coordinates adjusted to give N—H = 0.91 Å. All were included as riding contributions with isotropic displacement parameters 1.2 - 1.5 times those of the attached atoms.

### Crystal data

**Table:**

C_13_H_16_N_4_O_3_S_2_·H_2_O	*Z* = 2
*M**_r_* = 358.45	*F*(000) = 376
Triclinic, *P*1	*D*_x_ = 1.442 Mg m^−^^3^
Hall symbol: -P 1	Mo *K*α radiation, λ = 0.71073 Å
*a* = 9.0806 (12) Å	Cell parameters from 9913 reflections
*b* = 9.2444 (12) Å	θ = 2.4–29.2°
*c* = 10.7856 (14) Å	µ = 0.35 mm^−^^1^
α = 101.843 (2)°	*T* = 150 K
β = 100.1750 (19)°	Plate, colourless
γ = 105.9480 (19)°	0.26 × 0.26 × 0.12 mm
*V* = 825.48 (19) Å^3^	

### Data collection

**Table:**

Bruker SMART APEX CCD diffractometer	4292 independent reflections
Graphite monochromator	3773 reflections with *I* > 2σ(*I*)
Detector resolution: 8.3660 pixels mm^-1^	*R*_int_ = 0.034
φ and ω scans	θ_max_ = 29.2°, θ_min_ = 2.0°
Absorption correction: multi-scan (*SADABS*; Bruker, 2013)	*h* = −12→12
*T*_min_ = 0.83, *T*_max_ = 0.96	*k* = −12→12
15313 measured reflections	*l* = −14→14

### Refinement

**Table:**

Refinement on *F*^2^	0 restraints
Least-squares matrix: full	Hydrogen site location: mixed
*R*[*F*^2^ > 2σ(*F*^2^)] = 0.037	H-atom parameters constrained
*wR*(*F*^2^) = 0.099	*w* = 1/[σ^2^(*F*_o_^2^) + (0.0515*P*)^2^ + 0.3031*P*] where *P* = (*F*_o_^2^ + 2*F*_c_^2^)/3
*S* = 1.05	(Δ/σ)_max_ = 0.001
4292 reflections	Δρ_max_ = 0.44 e Å^−^^3^
210 parameters	Δρ_min_ = −0.37 e Å^−^^3^

### Special details

**Table:**

Geometry. Bond distances, angles *etc*. have been calculated using the rounded fractional coordinates. All su's are estimated from the variances of the (full) variance-covariance matrix. The cell e.s.d.'s are taken into account in the estimation of distances, angles and torsion angles
Refinement. Refinement on *F*^2^ for ALL reflections except those flagged by the user for potential systematic errors. Weighted *R*-factors *wR* and all goodnesses of fit *S* are based on *F*^2^, conventional *R*-factors *R* are based on *F*, with *F* set to zero for negative *F*^2^. The observed criterion of *F*^2^ > σ(*F*^2^) is used only for calculating -*R*-factor-obs *etc*. and is not relevant to the choice of reflections for refinement. *R*-factors based on *F*^2^ are statistically about twice as large as those based on *F*, and *R*-factors based on ALL data will be even larger.

### Fractional atomic coordinates and isotropic or equivalent isotropic displacement parameters (Å^2^)

**Table:**

	*x*	*y*	*z*	*U*_iso_*/*U*_eq_	
S1	1.14877 (5)	0.74276 (5)	0.62470 (3)	0.0316 (1)	
S2	0.40426 (4)	0.82259 (4)	0.00905 (3)	0.0218 (1)	
O1	0.50975 (12)	0.88281 (13)	0.30154 (9)	0.0263 (3)	
O2	0.16728 (13)	0.58384 (14)	0.15365 (11)	0.0325 (3)	
O3	0.38626 (12)	0.51837 (12)	0.14447 (9)	0.0244 (3)	
N1	0.91851 (13)	0.79013 (14)	0.46377 (11)	0.0200 (3)	
N2	1.05234 (16)	0.87115 (18)	0.92745 (12)	0.0330 (4)	
N3	0.73517 (14)	0.97498 (15)	0.76432 (11)	0.0257 (3)	
N4	0.73715 (13)	0.82181 (14)	0.30697 (10)	0.0204 (3)	
C1	0.98222 (15)	0.80180 (16)	0.58659 (13)	0.0193 (3)	
C2	0.92486 (15)	0.86183 (16)	0.69216 (12)	0.0192 (3)	
C3	0.79351 (15)	0.91523 (16)	0.66734 (12)	0.0194 (3)	
C4	0.72390 (15)	0.89929 (16)	0.53540 (12)	0.0207 (4)	
C5	0.79125 (15)	0.83911 (15)	0.44086 (12)	0.0185 (3)	
C6	1.1786 (2)	0.6781 (3)	0.46517 (17)	0.0480 (7)	
C7	0.99695 (16)	0.86718 (17)	0.82188 (13)	0.0225 (4)	
C8	0.60550 (15)	0.84739 (16)	0.24588 (12)	0.0194 (3)	
C9	0.59280 (16)	0.82956 (17)	0.10018 (13)	0.0215 (4)	
C10	0.28999 (17)	0.61849 (17)	−0.02404 (13)	0.0247 (4)	
C11	0.27024 (16)	0.57242 (17)	0.09924 (13)	0.0237 (4)	
C12	0.3870 (2)	0.4842 (2)	0.27094 (15)	0.0315 (5)	
C13	0.5180 (2)	0.4186 (2)	0.30413 (16)	0.0335 (5)	
O4	0.91965 (12)	0.72768 (13)	0.12641 (10)	0.0287 (3)	
H3A	0.79320	1.00160	0.84830	0.0310*	
H3B	0.65660	1.01610	0.74450	0.0310*	
H4	0.63290	0.92930	0.51240	0.0250*	
H4A	0.79970	0.79450	0.25550	0.0240*	
H6A	1.08460	0.59240	0.41090	0.0720*	
H6B	1.27060	0.64170	0.47300	0.0720*	
H6C	1.19680	0.76500	0.42460	0.0720*	
H9A	0.61410	0.73250	0.06270	0.0260*	
H9B	0.67560	0.91820	0.08960	0.0260*	
H10A	0.18450	0.59830	−0.08140	0.0300*	
H10B	0.34350	0.55330	−0.07120	0.0300*	
H12A	0.28430	0.40770	0.26670	0.0380*	
H12B	0.40330	0.58080	0.33910	0.0380*	
H13A	0.50470	0.32690	0.23340	0.0500*	
H13B	0.51520	0.38810	0.38570	0.0500*	
H13C	0.61990	0.49790	0.31490	0.0500*	
H4B	0.94980	0.77280	0.07090	0.0340*	
H4C	0.99220	0.69030	0.14630	0.0340*	

### Atomic displacement parameters (Å^2^)

**Table:**

	*U*^11^	*U*^22^	*U*^33^	*U*^12^	*U*^13^	*U*^23^
S1	0.0310 (2)	0.0527 (3)	0.0213 (2)	0.0314 (2)	0.0049 (1)	0.0089 (2)
S2	0.0225 (2)	0.0265 (2)	0.0184 (2)	0.0128 (1)	0.0009 (1)	0.0072 (1)
O1	0.0212 (5)	0.0418 (6)	0.0196 (5)	0.0182 (4)	0.0036 (4)	0.0063 (4)
O2	0.0282 (5)	0.0444 (7)	0.0334 (6)	0.0193 (5)	0.0132 (5)	0.0134 (5)
O3	0.0255 (5)	0.0313 (5)	0.0225 (5)	0.0142 (4)	0.0094 (4)	0.0108 (4)
N1	0.0184 (5)	0.0261 (6)	0.0182 (5)	0.0121 (4)	0.0041 (4)	0.0054 (4)
N2	0.0328 (7)	0.0520 (9)	0.0211 (6)	0.0253 (6)	0.0055 (5)	0.0097 (6)
N3	0.0227 (6)	0.0408 (7)	0.0167 (5)	0.0186 (5)	0.0037 (4)	0.0041 (5)
N4	0.0185 (5)	0.0299 (6)	0.0156 (5)	0.0132 (5)	0.0038 (4)	0.0053 (4)
C1	0.0171 (6)	0.0242 (6)	0.0188 (6)	0.0111 (5)	0.0032 (5)	0.0055 (5)
C2	0.0168 (6)	0.0253 (6)	0.0167 (6)	0.0098 (5)	0.0024 (4)	0.0054 (5)
C3	0.0159 (6)	0.0238 (6)	0.0180 (6)	0.0075 (5)	0.0030 (4)	0.0039 (5)
C4	0.0175 (6)	0.0281 (7)	0.0179 (6)	0.0119 (5)	0.0022 (5)	0.0047 (5)
C5	0.0158 (5)	0.0225 (6)	0.0168 (6)	0.0078 (5)	0.0010 (4)	0.0046 (5)
C6	0.0526 (11)	0.0851 (15)	0.0276 (8)	0.0544 (11)	0.0149 (7)	0.0128 (9)
C7	0.0190 (6)	0.0314 (7)	0.0202 (6)	0.0135 (5)	0.0053 (5)	0.0056 (5)
C8	0.0180 (6)	0.0226 (6)	0.0178 (6)	0.0088 (5)	0.0022 (4)	0.0050 (5)
C9	0.0190 (6)	0.0309 (7)	0.0190 (6)	0.0128 (5)	0.0047 (5)	0.0097 (5)
C10	0.0264 (7)	0.0266 (7)	0.0197 (6)	0.0094 (6)	0.0026 (5)	0.0045 (5)
C11	0.0223 (6)	0.0249 (7)	0.0235 (6)	0.0087 (5)	0.0043 (5)	0.0053 (5)
C12	0.0360 (8)	0.0431 (9)	0.0269 (7)	0.0198 (7)	0.0151 (6)	0.0184 (7)
C13	0.0391 (9)	0.0396 (9)	0.0301 (8)	0.0198 (7)	0.0100 (6)	0.0161 (7)
O4	0.0258 (5)	0.0376 (6)	0.0301 (5)	0.0171 (5)	0.0113 (4)	0.0116 (5)

### Geometric parameters (Å, º)

**Table:**

S1—C1	1.7550 (15)	C2—C3	1.415 (2)
S1—C6	1.7952 (18)	C2—C7	1.4208 (19)
S2—C9	1.7945 (15)	C3—C4	1.4111 (18)
S2—C10	1.8113 (16)	C4—C5	1.3776 (19)
O1—C8	1.2181 (18)	C8—C9	1.5262 (18)
O2—C11	1.2051 (19)	C10—C11	1.502 (2)
O3—C11	1.3436 (19)	C12—C13	1.499 (3)
O3—C12	1.4613 (19)	C4—H4	0.9500
O4—H4C	0.8400	C6—H6B	0.9800
O4—H4B	0.8400	C6—H6A	0.9800
N1—C1	1.3181 (18)	C6—H6C	0.9800
N1—C5	1.3543 (19)	C9—H9B	0.9900
N2—C7	1.1494 (19)	C9—H9A	0.9900
N3—C3	1.3414 (18)	C10—H10B	0.9900
N4—C5	1.4013 (16)	C10—H10A	0.9900
N4—C8	1.3639 (19)	C12—H12B	0.9900
N3—H3A	0.9100	C12—H12A	0.9900
N3—H3B	0.9100	C13—H13C	0.9800
N4—H4A	0.9100	C13—H13A	0.9800
C1—C2	1.4088 (19)	C13—H13B	0.9800
			
C1—S1—C6	101.26 (8)	O2—C11—C10	125.82 (14)
C9—S2—C10	101.89 (7)	O3—C12—C13	108.59 (14)
C11—O3—C12	115.32 (12)	C5—C4—H4	121.00
H4B—O4—H4C	102.00	C3—C4—H4	121.00
C1—N1—C5	116.87 (12)	S1—C6—H6A	109.00
C5—N4—C8	127.62 (12)	S1—C6—H6B	109.00
H3A—N3—H3B	120.00	H6A—C6—H6B	110.00
C3—N3—H3B	119.00	H6A—C6—H6C	109.00
C3—N3—H3A	119.00	S1—C6—H6C	109.00
C5—N4—H4A	116.00	H6B—C6—H6C	109.00
C8—N4—H4A	116.00	S2—C9—H9B	109.00
S1—C1—N1	119.69 (11)	C8—C9—H9A	109.00
S1—C1—C2	116.86 (10)	S2—C9—H9A	109.00
N1—C1—C2	123.45 (13)	H9A—C9—H9B	108.00
C1—C2—C3	119.22 (12)	C8—C9—H9B	109.00
C1—C2—C7	120.40 (13)	S2—C10—H10A	109.00
C3—C2—C7	120.38 (12)	C11—C10—H10A	109.00
N3—C3—C4	121.32 (13)	C11—C10—H10B	109.00
N3—C3—C2	121.69 (12)	H10A—C10—H10B	108.00
C2—C3—C4	116.98 (12)	S2—C10—H10B	109.00
C3—C4—C5	118.29 (13)	O3—C12—H12A	110.00
N1—C5—N4	110.75 (11)	C13—C12—H12A	110.00
N4—C5—C4	124.09 (13)	C13—C12—H12B	110.00
N1—C5—C4	125.16 (12)	O3—C12—H12B	110.00
N2—C7—C2	178.60 (17)	H12A—C12—H12B	108.00
O1—C8—C9	123.62 (13)	C12—C13—H13B	110.00
O1—C8—N4	123.88 (12)	C12—C13—H13C	109.00
N4—C8—C9	112.49 (12)	C12—C13—H13A	109.00
S2—C9—C8	114.23 (10)	H13A—C13—H13C	109.00
S2—C10—C11	111.95 (10)	H13B—C13—H13C	109.00
O3—C11—C10	110.84 (12)	H13A—C13—H13B	109.00
O2—C11—O3	123.32 (13)		
			
C6—S1—C1—N1	−0.35 (15)	S1—C1—C2—C7	−2.41 (19)
C6—S1—C1—C2	−179.63 (14)	N1—C1—C2—C3	−1.0 (2)
C10—S2—C9—C8	−83.84 (12)	N1—C1—C2—C7	178.34 (14)
C9—S2—C10—C11	63.79 (12)	C1—C2—C3—N3	−179.28 (14)
C12—O3—C11—O2	−4.3 (2)	C1—C2—C3—C4	2.2 (2)
C12—O3—C11—C10	174.39 (12)	C7—C2—C3—N3	1.4 (2)
C11—O3—C12—C13	177.43 (13)	C7—C2—C3—C4	−177.10 (14)
C5—N1—C1—S1	−179.33 (11)	N3—C3—C4—C5	179.07 (14)
C5—N1—C1—C2	−0.1 (2)	C2—C3—C4—C5	−2.4 (2)
C1—N1—C5—N4	179.28 (13)	C3—C4—C5—N1	1.5 (2)
C1—N1—C5—C4	−0.2 (2)	C3—C4—C5—N4	−177.88 (13)
C8—N4—C5—N1	174.69 (14)	O1—C8—C9—S2	−13.0 (2)
C8—N4—C5—C4	−5.9 (2)	N4—C8—C9—S2	168.00 (10)
C5—N4—C8—O1	−3.9 (2)	S2—C10—C11—O2	86.96 (18)
C5—N4—C8—C9	175.09 (13)	S2—C10—C11—O3	−91.73 (13)
S1—C1—C2—C3	178.25 (11)		

### Hydrogen-bond geometry (Å, º)

**Table:**

*D*—H···*A*	*D*—H	H···*A*	*D*···*A*	*D*—H···*A*
N3—H3*A*···N2^i^	0.91	2.43	3.3291 (18)	168
N3—H3*B*···O1^ii^	0.91	2.03	2.9345 (18)	177
N4—H4*A*···O4	0.91	2.01	2.9212 (16)	174
O4—H4*B*···N2^iii^	0.84	2.17	3.0032 (19)	172
O4—H4*C*···O2^iv^	0.84	2.09	2.9122 (18)	168
C4—H4···O1	0.95	2.25	2.8484 (17)	121

Symmetry codes: (i) −*x*+2, −*y*+2, −*z*+2; (ii) −*x*+1, −*y*+2, −*z*+1; (iii) *x*, *y*, *z*−1; (iv) *x*+1, *y*, *z*.
